# The development and function of CD11c^+^ atypical B cells - insights from single cell analysis

**DOI:** 10.3389/fimmu.2022.979060

**Published:** 2022-08-22

**Authors:** Xin Gao, Ian A. Cockburn

**Affiliations:** Division of Immunology and Infectious Disease, John Curtin School of Medical Research, The Australian National University, Canberra, ACT, Australia

**Keywords:** B cells, atypical B cell, single cell RNA seq, malaria, HIV, autoimmunity, SLE

## Abstract

CD11c^+^ T-bet^+^ atypical B cells (ABCs) have been identified in the context of vaccination, acute and chronic infections and autoimmune disease. However, the origins and functions of ABCs remain elusive. A major obstacle in the study of ABCs, and human MBCs more generally, has been the use of different phenotypic markers in different contexts to identify what appear to be phenotypically similar cells. Advances in single-cell RNA sequencing (scRNA-seq) technology have allowed researchers to accurately identify ABCs in different immune contexts such as diseases and tissues. Notably, recent studies utilizing single cell techniques have demonstrated ABCs are a highly conserved memory B cell lineage. This analysis has also revealed that ABCs are more abundant in ostensibly healthy donors than previously thought. Nonetheless, the normal function of these cells remains elusive. In this review, we will focus on scRNA-seq studies to discuss recent advances in our understanding about the development and functions of ABCs.

## Introduction

Memory B cells form after infection or vaccination in order to provide a rapid anamnestic response to reinfection. These cells in humans are characterized by the high expression of CD27 and CD21 (1). As such the markers and function of these so-called classical memory B cells (cMBCs) are well understood. However, a now seminal study identified a population of B cells expressing FCRL4 but not CD27 in the tonsils. This novel population was classified as a memory cell because of negligible BCL6 and BLIMP1 expression, the hallmark transcription factors (TFs) of germinal center (GC) B cells and plasma cells (PC) respectively (2). Because of their identification in the tonsils these cells were initially designated as tissue-like memory B cells, however their function was unknown. The subsequent identification of similar populations mostly in conditions of autoimmunity, chronic infection and aging but without functional characterization has led to these cells being grouped under the umbrella of atypical B cells (ABCs) which is the terminology we will use here.

The study of ABCs has been hampered by a lack of consistent flow cytometry markers to identify mouse and human ABCs. Additionally, the appearance of ABCs in many different contexts such as infection and autoimmunity has made it harder to understand what drives the development of ABCs and their function. Recently scRNA-seq has been used to investigate the B cell compartment in a variety of human samples including blood, tonsil, lymph node, bone marrow, kidney, liver, synovial tissues and cerebrospinal fluid (3–14). Intriguing, most of these datasets have identified ABCs as a distinct subset by unsupervised clustering indicating their conserved presence in B cell compartment. These studies have also contributed insights into the origin and function of ABCs which we will describe here.

## The identification of ABCs

The early analyses of ABCs in the tonsils revealed that these cells were enlarged with low CD21 expression indicative of an activated phenotype (2). Additionally, ABCs had a distinct integrin and chemokine receptor expression profile, characterized by the expression of CD11c and loss of CXCR5, potentially altering their migratory patterns and tissue localization (2, 15). Finally, while they were classified as memory B cells based on the lack of BLIMP1 and BCL6 expression, they also had a distinct transcription factor profile compared to cMBCs as they upregulated RUNX1, RUNX2 and SOX5 expression, suggesting they likely maintain a distinct global transcriptomic profile instead of being an unstable or transient stage of memory B cell development (15).

**Figure 1:**
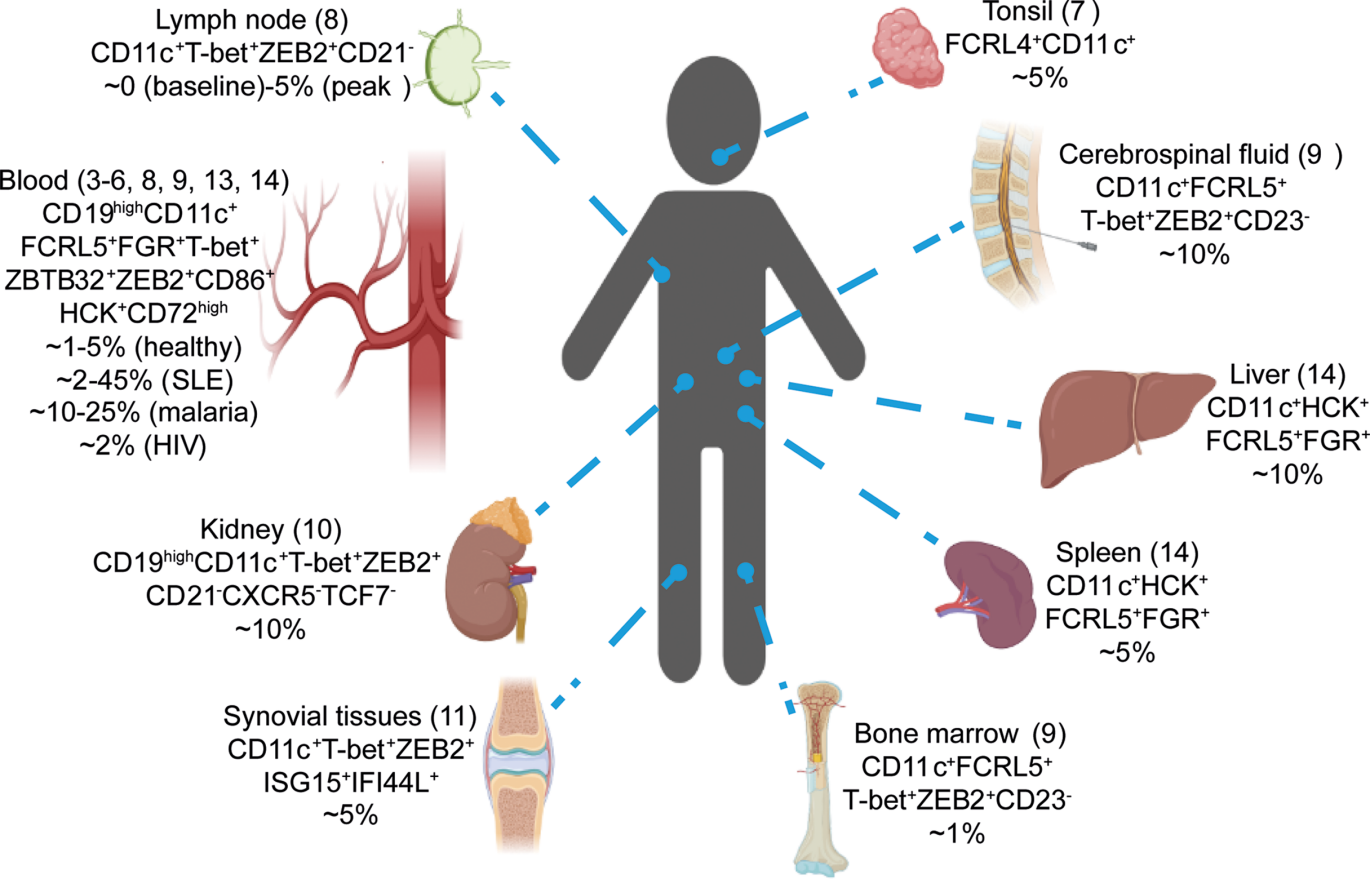
scRNA-seq revealed the conserved presence of ABCs in human. Single-cell RNA-seq studies have identified ABCs in numerous tissues by unsupervised clustering (3-14). Tissues, signature genes and estimated percentages in total B cells for ABCs cluster are shown.

Subsequently, ABCs were also reported in human blood (in healthy individuals and patients with infections or autoimmunity) and other tissues including spleen, lymph nodes, bone marrow, kidney, liver, synovial tissues, cerebrospinal fluid and choalveolar lavage fluid (3–14, 16–29) (**Figure 1**). Interestingly, ABCs preferentially located in the blood, spleen and bone marrow, with fewer cells detected in lymphoid systems such as lymph nodes, thoracic duct fluid and tonsils (28, 29). More recently ABC-like populations have also been identified in mice facilitating experimental studies. Initially a population of CD11c^+^CD11b^+^ or CD21^-^CD23^-^ cells was identified in older mice and classed as age-associated B cells (30, 31). Subsequent studies in ageing, infection and autoimmunity models have shown that murine CD11c^+^CD11b^+^ B cells are phenotypically and transcriptomically similar to human ABCs as they upregulate *Cd72, Hck*, *Tbx21*, *Zeb2* and *Zbtb32* (30–38). Consistent with the preferential tissue localization of human ABCs, mouse hemagglutinin-specific CD11c^+^T-bet^+^ ABCs were preferentially maintained in the spleen but not the lymph nodes after Influenza virus infection, but they could not migrate from the spleen of primary infected mice into conjoined naïve mice in parabiosis experiments (28). Using Bcl6-deficient mice, a recent study suggested that ABCs form in the extrafollicular compartment and migrate to the marginal zones of the spleen potentially explaining their preference for this compartment (39).

Historically ABCs have been identified using flow cytometry based on a handful of key markers. Lack of CD27 and CD21 has been frequently used to identify human ABCs during chronic or repeated infection with *Plasmodium* (16). However, a more recent study has demonstrated that CD21^-^CD27^-^ circulating B cells could be further separated into FCRL5^-^ and FCRL5^+^ subsets, and only the FCRL5^+^ subsets expressed ABC-associated signature genes whereas the FCRL5^-^ subset resembled classical memory/naive cells (40). Similar concerns were also raised in mouse studies. For example, CD21^-^CD23^-^ B cells were identified as ABCs in aging mice whereas these cells have been shown in a surrogate light chain deficient lupus mouse model to be unlikely to be ABCs because of their negligible CD11c expression (41). CD11c has been used as a canonical ABC marker in most ageing, infection and autoimmunity models (31–38). Thus, the sensitivity and specificity of any gating strategy designed to identify ABCs is unclear. Moreover, a gating strategy that efficiently identifies ABC-like cells in one context may not identify transcriptionally similar cells in another setting.

Because single cell RNA-seq relies on the expression of thousands of genes to classify and cluster cells, it should circumvent the problems of identification by flow cytometry which is limited to at most 10s of markers. Thus, single cell RNA-seq has the potential to aid the identification of more universal markers of the ABC population and determine if cells identified in different contexts are in fact phenotypically similar to each other. Notably, nearly all datasets have detected ABCs by unsupervised clustering regardless of the tissue studied, frequently marked by genes such as FCRL4^+^ FCRL5^+^ CD11c^+^ T-bet^+^ ZEB2^+^ and FGR^+^ (**Figure 1**) (3–13). Similar observations had been reported by comparing ABCs in different disease contexts. By integrating the scRNA-seq datasets obtained side-by-side from healthy individuals, malaria and HIV infected patients, one recent study showed ABCs from these donors co-localized into one cluster suggesting they had overlapping transcriptomes (13). Further analysis comparing ABC signature genes derived from different datasets demonstrated the high similarity between circulating ABCs induced by infection and autoimmune diseases such as systemic lupus erythematosus (SLE), rheumatoid arthritis and common variable immunodeficiency (13). Interestingly, a recent scRNA-seq dataset on horse blood also reported a ABCs cluster marked by the expression of FCRL4, T-bet and CD11c (42). These results suggested that ABCs identified in different tissues, organisms and in healthy individuals and patients with different diseases, were highly conserved and shared a similar global transcriptomic profile.

Given that ABCs are highly conserved in different contexts, an outstanding question is the identity of the best markers to identify ABCs. We attempted to address this using cellular indexing of transcriptomes and epitopes by sequencing (CITE-seq) (43). In this approach, antibodies conjugated to oligonucleotide sequences (barcodes) can be used to label cells in a single cell experiment and the relative levels of barcode sequence used to measure the level of surface expression. Using barcoded antibodies against CD21, CD27, CXCR3 and CD11c to phenotype ABCs in *Plasmodium*-exposed donors and healthy individuals, we discovered that although ABCs in *Plasmodium*-exposed donors were predominantly CD21^-^CD27^-^, ABCs in healthy individuals commonly expressed CD21 and CD27, suggesting these markers were not optimal for identifying the ABC lineage. In contrast, CD11c was upregulated on ABC clusters in all subjects, suggesting CD11c^+^ was a more useful marker for the ABC population (3). Nonetheless the variable expression of all ABC markers probably leads to an undercount of these cells in healthy individuals. Analysis of a more extensive panel of antibodies should in theory yield markers with even better resolution. However, our finding that CD11c is perhaps the most useful marker for ABCs is consistent with a recent cytometry by time of flight (CyTOF) study comparing 351 surface molecules on human circulating B cells which reported CD11c as a defining marker for ABCs in healthy individuals (44). Overall, published scRNA-seq studies showed CD11c, FCRL4 and FCRL5 were the most frequently up-regulated surface genes for ABCs cluster, accompanied by TFs T-bet and ZEB2 (3–14). The utility of these markers in mice is unclear, CD11c is commonly used but mouse Fcrl5 is not a direct homolog of the human protein of the same name and appears to be a marker of all murine memory B cells (32, 38).

Despite the highly conserved features of ABCs among different immunological contexts, our previous scRNA-seq analysis suggested that ABCs were heterogenous and had different degrees of polarization towards the atypical phenotype. In our CITE-seq analysis we found CD21^-^CD27^-^ ABCs which predominated in the *Plasmodium-*exposed individuals expressed the highest levels of ABC-associated markers such as CD11c and T-bet, whereas ABCs in healthy individuals were predominately CD21^+^CD27^+^CD11c^int^ (3). Longitudinal analysis of B cells specific for the *Plasmodium* circumsporozoite protein in previously malaria-naïve individuals receiving a whole parasite vaccine revealed that CD11c^+^ ABCs were induced in primary immunization but with repeated immunizations these cells cell increasingly adopted a CD21^-^CD27^-^ phenotype (3). Additionally, FCRL4 and FCRL5 have distinct expression patterns on ABCs under different immunological contexts. Although FCRL4 was highly expressed on circulating CD21^-^CD27^-^ ABCs in HIV infected patients, it was not expressed by circulating CD21^-^CD27^-^ ABCs in *Plasmodium*-infected patients nor CD11c^+^CXCR5^-^ ABCs in SLE patients (20, 22, 23).

## The development of ABCs

While single cell technologies have shown that ABCs form a distinct lineage found not only in disease settings but also in healthy individuals and protective immune responses, these techniques can also help to identify the development and origin of ABCs at the cellular level, the extracellular stimulus level and the TF level.

A key controversy has been whether ABCs have a GC dependent or independent origin. BCR sequence analysis in multiple settings has shown that ABCs have comparable or slightly lower levels of somatic hypermutation (SHM) with cMBCs and such values were much higher than naïve or follicular B cells in humans and mice (3, 7, 13, 28, 45–48), suggesting ABCs are GC-experienced cells. Similar observations also reported on Influenza-specific ABCs which were also mutated but formed distinct clades within phylogenetic trees compared to cMBCs and plasmablasts (49). Consistent with this, B cell specific knock-out of *Bcl6* abolished the development of CD11c^+^ T-bet^+^ ABCs in mice upon *E. muris* infection (36). However, other evidence suggests a GC-independent origin for ABCs. IgD^+^ cells with the features of ABCs have been reported in SLE patients and in aged female mice (23, 30, 31), while *Bcl6* in B cells was not required for CD11c^+^ T-bet^+^ ABCs formation in Lymphocytic Choriomeningitis Virus (LCMV) and Influenza virus infection in mice (39).

Regardless of the GC or non-GC origin of ABCs their formation appears to be T dependent. Several reports have shown that inhibiting T-B interactions by knocking-out MHC-II, CD40 or CD40L completely abolishes ABC formation in aged, lupus-prone and *E.muris* infected mice (34, 36, 46). Whether follicular helper T (Tfh) cells are required for ABC-formation remains controversial since contradictory results have been reported using T cell-specific *Bcl6* deletion in LCMV and *E.muris* infection models (36, 39). Nevertheless, these studies suggest that ABC formation occurs at the T-B border, rather than in GC structures though this localization may still permit somatic hypermutation to occur, explaining the BCR mutations present in ABCs. Of note, ABCs could also be GC independent, but carry high levels of SHM if they arose from GC-experienced cMBCs. This idea was supported by the observation that secondary vaccination or infection can induce stronger CD11c^+^ ABC production than the primary response (3, 17). Nevertheless, perhaps the best theory to generalize these observations is that the origin of ABC is not restricted to a specific type of B cell. Naïve, GC B cells and cMBC might all be the source of ABC as long as they receive the right extracellular stimulus. This idea is supported by the observation that ABCs in *Plasmodium-*exposed Malian children could be separated into IgD^-^IgG^+^, IgD^+^IgM^+^ and IgD^+^IgM^low^ subsets with SHM rates equivalent, respectively, to cMBCs (suggesting GC and cMBC origin), naïve B cells (suggesting a naïve B cell origin) and intermediate between naïve and cMBC (suggesting T-B border origin) (13).

Extracellular factors may also play a key role in the formation of ABC. Our previous study looking at ABCs in individuals with high and low levels of malaria exposure found that not only were *Plasmodium*-specific ABCs more common in people from high transmission areas, but bystander B cells specific for tetanus toxin were also more likely to be ABCs (50). Thus, inflammatory mediators may drive ABC formation. According to two pioneer studies, ABCs were highly responsive to TLR7 and TLR9 ligand stimulation *in vitro*, and chronic TLR7 but not TLR9 ligand treatment can induce the formation of CD11c^+^CD11b^+^ ABCs *in vivo* (31). Consistent with this, B cell specific deletion of *Tlr7* or *Myd88* has been shown to abolish the development of ABCs in aged female mice (31), while malaria induced ABC-generation was slightly impaired when *Tlr9* was specifically knocked-out in B cells (51).

In addition to TLR signaling, cytokines also play a central role in regulating ABC formation. IL-21 was found to be crucial for ABC development in the SWAP-70 and DEF6 double knock-out (DKO) model of lupus as well as the *E.muris* infection model (36, 52). IL-21 transgenic mice also have spontaneous ABC formation (53), while IL-21 combined with TLR7 or TLR9 ligands, or with anti-CD40 plus anti-BCR antibodies, induced the differentiation of CD11c^+^T-bet^+^ cells *in vitro* (23, 52–54). In addition to IL-21, IFN-γ has been shown to promote the generation of CD11c^+^T-bet^+^ ABCs upon Influenza and *E.muris* infection as well as in a WAS chimera lupus model (34, 36, 53). However, IFN-γ does not induce CD11c expression in human and mouse B cells in *in vitro* culture (53, 55). IL-4 has been shown to suppress the expression of CD11c and T-bet in mouse B cell culture and upon *H. polygyrus* infection *in vivo* (23, 53).

T-bet is often considered a key transcription factor for ABC formation. Although T-bet is one of the highest expressed TFs in ABCs, and T-bet overexpression can promote CD11c expression (37), studies have shown that T-bet knock-out on B cells does not affect the generation of CD19^high^CD11c^+^ ABCs upon *E. muris* infection or in WAS chimera lupus mice (34, 36). RUNX1 and RUNX2 overexpression also does not promote FCRL4 expression although both genes are highly expressed by human ABCs (15). Irf5 has also been reported to be required for ABC formation in the SWAP-70 and DEF6 double knock-out lupus model (52). However, to date, the master regulatory TFs for ABCs’ development have not been identified. Published transcriptomic datasets had reported several candidates such as ZBTB32, SOX5, BHLHE40, TOX and ZEB2 that were upregulated in human and mouse ABCs (20, 38), especially ZEB2 which has been proposed to control ABC formation in lupus (56).

## The function of ABCs

Perhaps the major outstanding question in ABC biology is the role these cells play in the immune system. scRNA-seq datasets suggest that ABCs sit with the memory/naïve super-cluster but are distinct from cMBCs, activated, and pre-GC memory B cells (7). Consistent with this, trajectory analysis of circulating B cells shows that ABC form a stand-alone developmental branch sharing a common progenitor with cMBCs (3, 13). These observations suggest ABCs might have a different function from well-studied subsets like cMBCs. Previous studies have reported many functional observations on ABCs which have posited three possible roles for ABCs:1) ABCs are exhausted cells 2) ABCs are pre-plasma cells and 3) APCs are specialized for antigen presentation.

Having been initially identified in a variety of pathological conditions ABCs were assumed to be an exhausted or non-functional population. In particular early experiments showed that ABCs were unresponsive to BCR/CD40 stimulation and *in vitro* analysis reported that ABCs had similar or slightly weaker capacity to differentiate into PC compared with cMBCs (23–25, 30, 31, 40, 54). Using probes to track CSP-specific CD11c^+^ ABCs after vaccination we found that B cells peaked around 2 weeks after malaria vaccination (~30%) in human, but this number dropped to baseline (~10%) after 6 weeks, suggesting that ABCs were not as capable as cMBCs in surviving through the memory contraction phase (3). Consistent with this, CD19^high^ human ABCs were more prone to FasL induced apoptosis than cMBCs (45). Additionally, the numbers of circulating ABCs dropped significantly after the resolution of malaria in humans and mice or after ART treatment in HIV patients (17, 21, 32, 38). This evidence supported the notion that ABCs were “exhausted-like” cells which may act as a non-functional memory lineage to antagonize strong humoral responses during chronic infections and autoimmunity. A variant of the “exhausted cell” hypothesis is that ABCs are formed in conditions of stress and represent a better-than-nothing emergency response. In support of this a recent study showed that B cell responses to a COVID vaccine in individuals given immunosuppressive drugs were predominantly of an ABC phenotype (57).

A second hypothesis for ABC function is that they are poised to develop into plasma cells. While the inability of ABCs to produce antibody in response to BCR stimulation may seem to contradict this, ABCs have been shown to be responsive to TLR7 ligands (30, 31). Moreover, previous studies suggested ABCs in SLE patients express higher levels of PC transcription factors IRF4 and BLIMP, hinting that they are PC-precursors, capable of differentiating in to autoantibody secreting cells (23). Consistent with this, ABCs in SLE patients were found to be highly enriched with auto-reactive B cell clones while vaccine-induced ABCs were enriched for Influenza-specific clones (49, 54). In animal models *in vivo* depletion of ABCs by a B cell specific CD11c-DTR system, significantly reduced the generation of autoantibody in TLR7-ligand chronically treated mice (31). However, a serious technical issue is whether CD11c-DTR system exclusively depleted ABCs without affecting other B cell populations because this system has been used to deplete general activated B cells including GC B cells (58, 59). Moreover, if ABCs were poised to differentiate into PCs, we would expect to observe cells in intermediate stages of differentiation by pseudotime analysis of single cell data. However, scRNA-seq trajectory analysis on tonsillar B cells does not reveal that ABCs are closer to the PC cluster than other memory B cell subsets (7). Nevertheless, ABCs do retain the potential to differentiate into PCs, since adoptively transferring ABCs from pre-immunized mice could potentiate antibody secretion in recipient mice upon Virus Like Particle (VLP) immunization or LCMV infection (39, 48).

Finally, ABCs have been proposed to be specialized for antigen uptake and presentation to T cells. Both flow cytometry, sc-RNA seq and conventional RNA seq show that ABCs upregulate antigen presentation genes including MHC-II and CD86 (13, 31, 38, 54). Interestingly, CD19^hi^ T-bet^+^ ABCs in healthy and HIV infected individuals preferentially localized in non-GC and extrafollicular zones in LNs (45), and CD19^+^B220^+^CD11c^+^ ABCs in aged mouse preferentially localized at the T-B border in the spleen (60), implying that ABCs are specialized to interact with T cells. Consistent with this, non-specific CD11c+ ABCs were found to be better at presenting OVA-protein to OT-II cells than follicular B cells (60). Another interesting finding was that ABCs were better than follicular B cells at priming OT-II cells into CXCR5^+^ follicular helper T (Tfh)-like cells in *in vitro* co-culture, while ABC generation preceded Tfh expansion in the CD19^cre^ Ship^fl/fl^ lupus model (35). Notably, a recent study showed that human CD21^-^CD27^-^ ABCs had enhanced BCR signaling and antigen uptake capacity to plate bound antigen (anti-λ/κ bound to plasma membrane sheets) (61). Further work is required to understand the role of ABCs in antigen presentation– it may be that ABCs present antigen to prime a particular T cell fate (e.g. Tfh vs Th1), or conversely they may have a regulatory role sequestering antigen and limiting antigen presentation.

## Concluding remarks

Conventional flow cytometry has struggled to accurately identify ABCs in different immune contexts. Emerging scRNA-seq technologies however have overcome such limitations and significantly advanced our understanding of ABC biology. First, the simultaneous detection of thousands of genes has allowed us to identify ABCs in various immune contexts and demonstrated their conserved and heterogenous properties between tissues, organisms and diseases. ABCs almost uniformly express CD11c, T-bet and ZEB2 but have different usage of FCRL4 vs FCRL5 depending on the immune context. Secondly, the use of CITE-seq has allowed us to explore better markers for ABCs, demonstrating that CD11c expression is more useful than CD21^-^CD27^-^ to detect the ABC population in healthy individuals. Third, trajectory analysis has shown that ABCs form a separate developmental branch to well-established B cell lineages like activated B cells and cMBCs, suggesting that the formation of ABCs requires distinct extracellular stimuli and that their functions are likely to be different from these well-studied subsets.

Despite these advances, we still have limited knowledge on the origin and normal functions of ABCs. Research in these areas has been hampered by the inconsistent observations between different animal models, the lack of a reliable systems to specifically knock-out ABCs and the lack of antigen-specific models to generate sufficient cells for functional analysis. Therefore, future studies should focus on using antigen specific models to identify the master TFs for ABCs, and potentially use these mice as a tool to specifically knock-out ABCs and thus study their functions in normal and pathogenic settings.

## Author contributions

XG wrote the original draft of the manuscript. IC edited the manuscript. Both authors reviewed and approved the final version.

## Acknowledgments

Xin Gao is supported by a PhD studentship from the Australian National University. Work in the Cockburn Lab is supported by an NHMRC Investigator Grant to IC (GNT2008648). The funders had no role in the drafting of the manuscript or the decision to publish.

## Conflict of interest

The authors declare that the research was conducted in the absence of any commercial or financial relationships that could be construed as a potential conflict of interest.

## Publisher’s note

All claims expressed in this article are solely those of the authors and do not necessarily represent those of their affiliated organizations, or those of the publisher, the editors and the reviewers. Any product that may be evaluated in this article, or claim that may be made by its manufacturer, is not guaranteed or endorsed by the publisher.
